# Supplementary figures and images for: Wearable sensor-based performance status assessment in cancer: A pilot multicenter study from the Alliance for Clinical Trials in Oncology (A19_Pilot2)
Source: PLOS Digit Health. 2023 Jan 26;2(1):e0000178. doi: 10.1371/journal.pdig.0000178 (PMC9931326; doi:10.1371/journal.pdig.0000178)

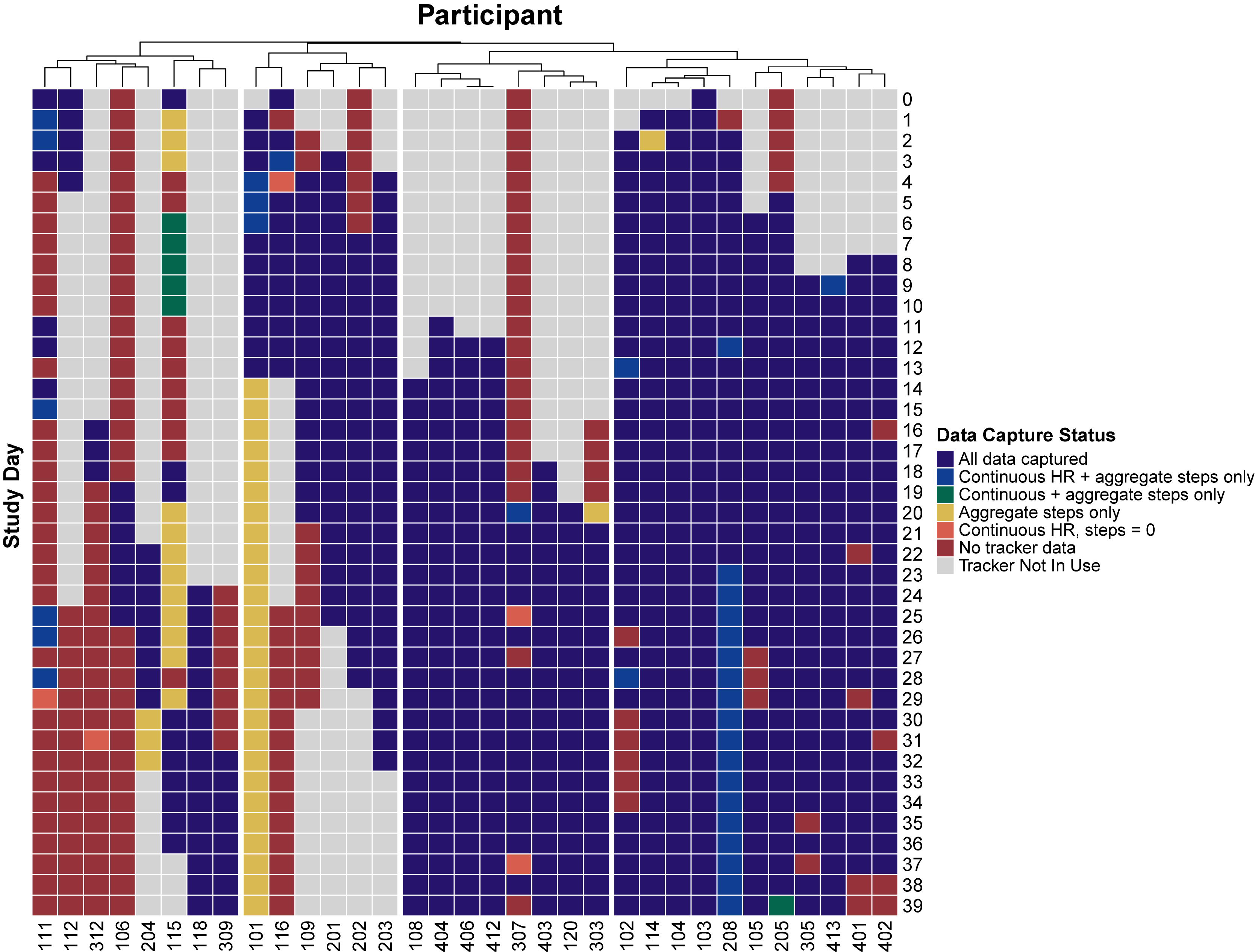

Supplement: S1 Fig — (TIF) [file pdig.0000178.s002.tif]

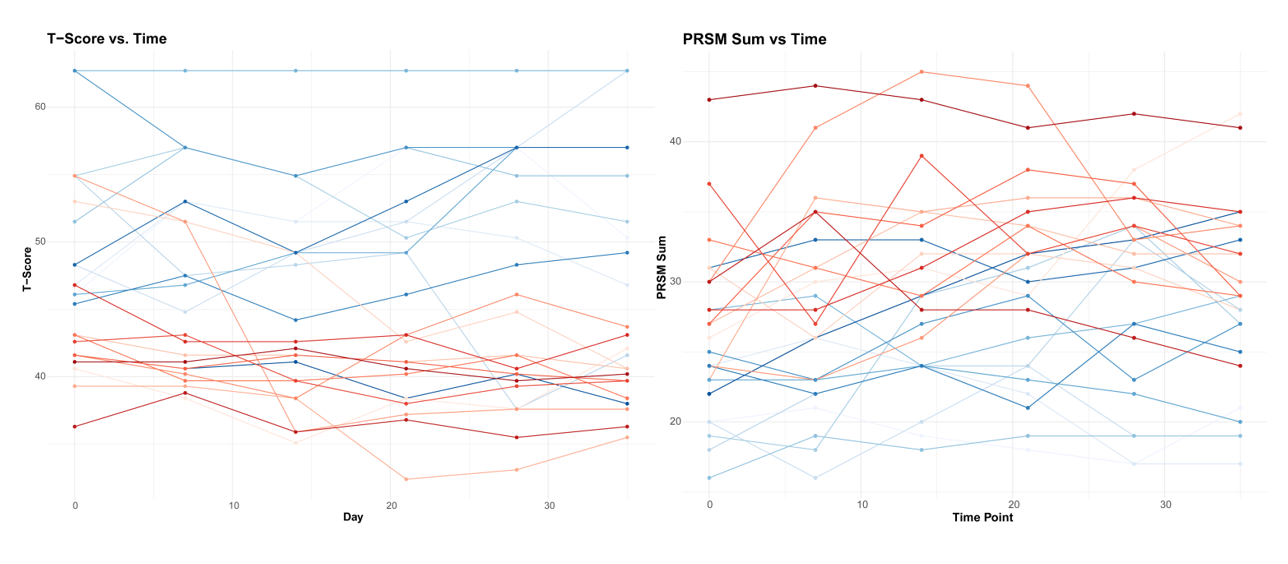

Supplement: S2 Fig — S2a Fig. Longitudinal Physical Function T-score assessments (original cohort). Longitudinal analysis of T-Score vs days since baseline survey completion. Hierarchical clustering revealed two distinct groups of patients, with low and high PROMIS Physical Function outcomes, designated by color schemes. Some participants maintained consistent results while others deviated between weeks. Only participants with data for all time periods were included in this figure. Higher T-Scores are associated with improved performance. S2b Fig. Longitudinal symptom burden (original cohort). Hierarchical clustering of symptom burden revealed two participant cohorts with low and high symptom burdens, as per the respective blue and red color schemes. The spaghetti plot below depicts total symptom burden scores in relation to days since baseline survey completion. Only participants with PRSM sums for every week were included. Higher PRSM sums are associated with increased symptoms. (TIF) [file pdig.0000178.s003.tif]

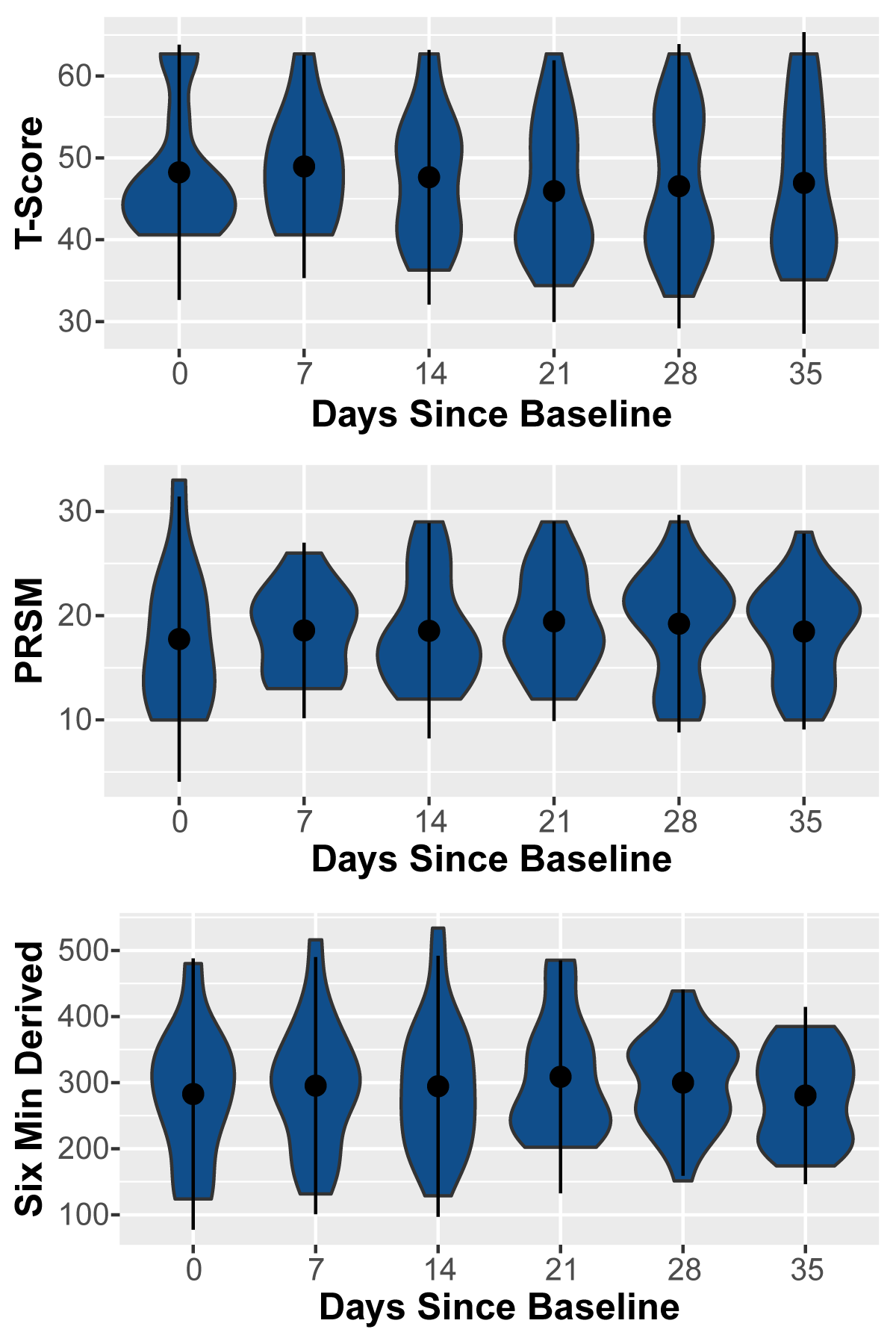

Supplement: S3 Fig — (TIF) [file pdig.0000178.s004.tif]

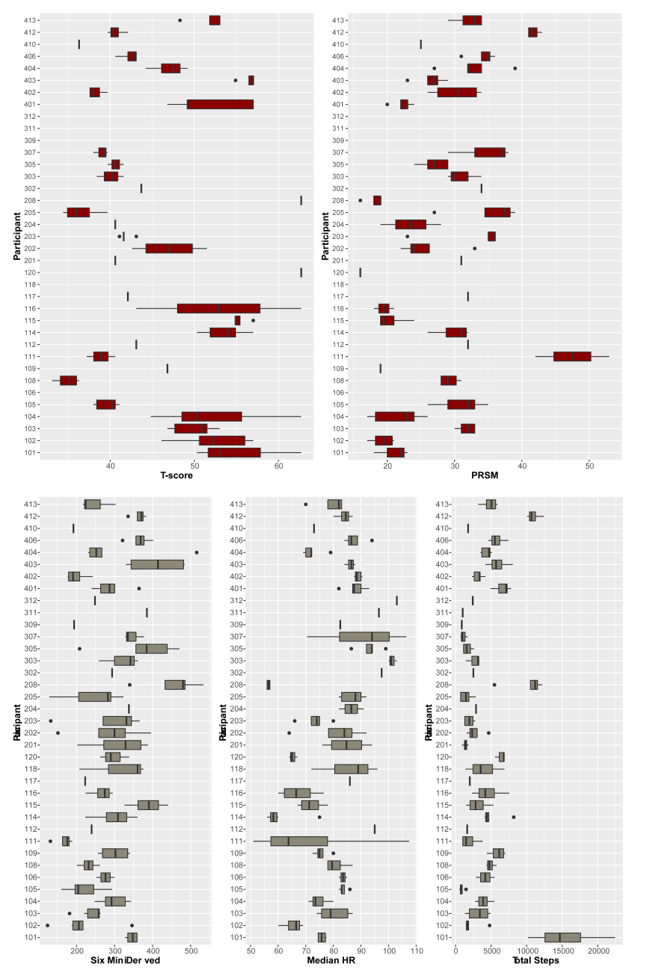

Supplement: S4 Fig — (TIF) [file pdig.0000178.s005.tif]

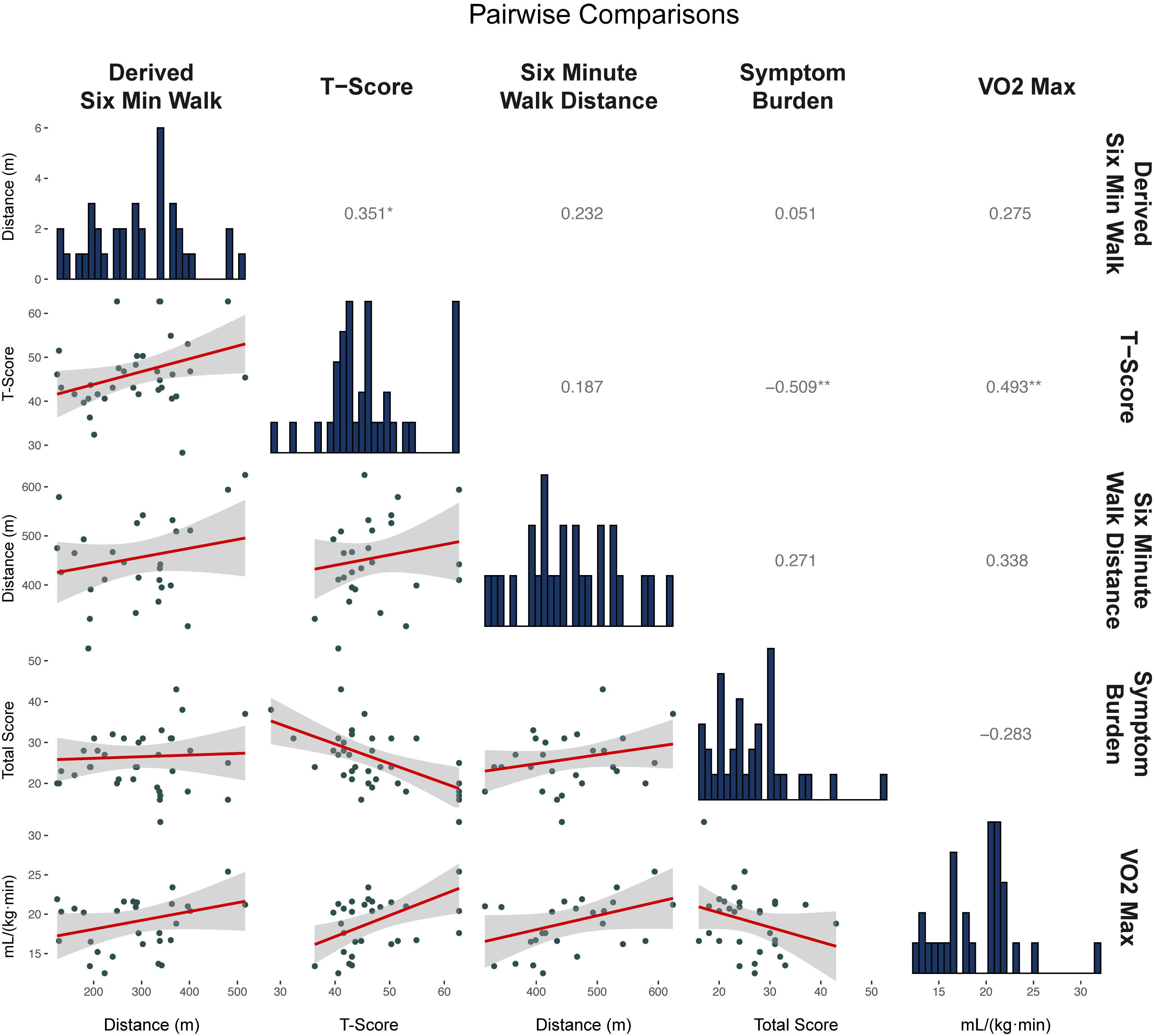

Supplement: S5 Fig — (TIF) [file pdig.0000178.s006.tif]

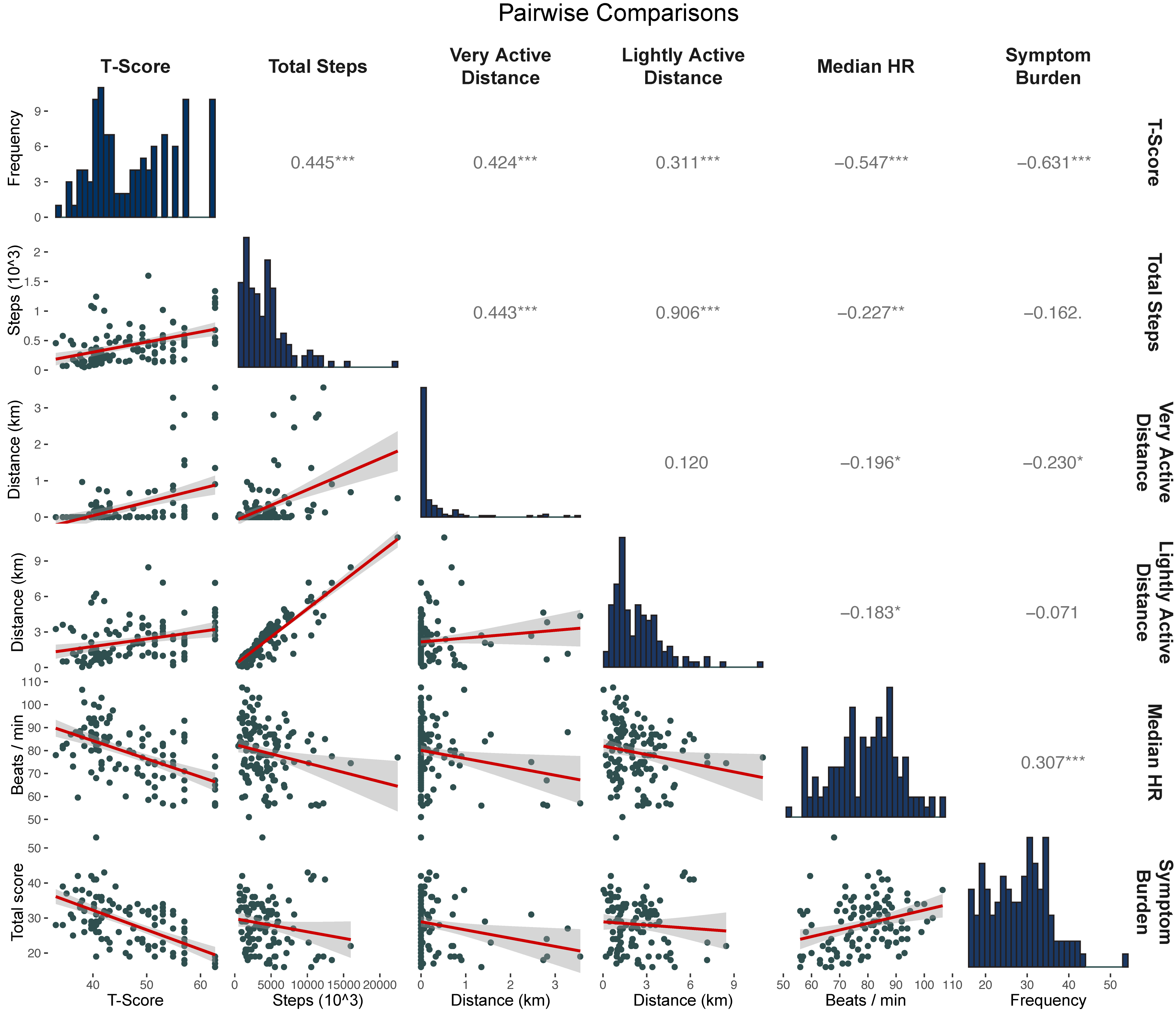

Supplement: S6 Fig — (TIF) [file pdig.0000178.s007.tif]
