# Supplementary material for: Wearable sensor-based performance status assessment in cancer: A pilot multicenter study from the Alliance for Clinical Trials in Oncology (A19_Pilot2)
Source: PLOS Digit Health. 2023 Jan 26;2(1):e0000178. doi: 10.1371/journal.pdig.0000178 (PMC9931326; doi:10.1371/journal.pdig.0000178)
Supplement: Attachment — Submitted filename: reviewer_response plos digital health 10.1.22.docx [file pdig.0000178.s011.docx]

To the Editors:

We thank the reviewers for their time and insightful comments and suggestions. Below we address the points raised by the reviewers (text indicated in blue italic font) and indicate how we have updated the manuscript.

--

**Response to reviewers:**

Reviewer #1

*Major Concerns
•    The methods and results sections feel disorganized and out of sync with each other and with the stated and preregistered aims of the study.  If the primary objective was to assess the feasibility of this measurement approach, feasibility should be better defined, and analyses that are not central to the aims of this work omitted.*

We thank the reviewer for drawing our attention to this. Originally, our abstract was separated into introduction, methods, results, and discussion sections. We have combined and simplified these into a unified abstract and emphasized the feasibility aspect of the study – which was the pre-specified primary objective. Feasibility in the protocol was defined as meeting 75% of the following metrics:

- Proportion of patients who complete CPET and 6MWD test without significant adverse events
- Proportion of recruited patients who wear the FitBit for at least 8 hours per day
- Proportion of patients for whom all data is successfully recorded within the UNC PRO-Core database and Fitabase.

In this context, baseline exercise testing was not feasible (68%) while the other metrics were.

Of note, we have made the FitBit feasibility more stringent in the abstract than originally described since usable data (84%) does not always result from wearing the FitBit device (battery failure, sync failure, data compression due to low sync frequency, etc.).

Additionally, we have added the original study objectives within the ‘Study Design’ portion of the Methods section.

The percentage of screened patients enrolling (81%) was also added to the Results section under ‘Data collection’.

*For example, what is the purpose of comparing baseline CPET and baseline 6MWT?*

We do not directly compare baseline CPET and 6MWT in the body of the manuscript (although it is included as a subpanel in Figure S5 for the sake of completeness). We suspect the reviewer is asking why we compared our sensor derived 6MWT to the actual 6MWT. Our intent was to expand the scope of ‘feasibility’ as the data allows.

To clarify our intent in the flow of the results section, we moved the paragraph entitled ‘Hierarchical clustering …’ immediately below the ‘Data collection’ setting, created a separate section entitled ‘Data completeness…’ immediately after, which is followed by ‘Physical performance testing…’ which the reviewer is referencing. Given the updated order, we hope this emphasizes the thread of feasibility of tracker usage from multiple different domains.

*Since validation of sensor metrics was not named as a goal, why compare tracker-derived sleep data to self-reported sleep data?*

We appreciate this insightful question and agree that validation of the sensor (and survey) metrics was not a goal of the study. As a feasibility study, our goal is to guide future studies and we felt the inclusion of these summary data will help guide future research.

*What was the purpose of the hierarchical clustering of individual items/Figures 3 and 4?*

Our intent as with the sleep metric was to explore the instruments used and guide future studies under the broader umbrella of ‘feasibility’. Since these instruments are widely used, we felt it was helpful to identify the critical components of these instruments in our cohort.

*•    Given the introduction’s initial focus on clinician-assigned performance status, I expected the paper to consider how patient-generated data related to a more traditional clinician-based measure.  Was clinician-rated ECOG PS or KPS assessed?  How does ECOG or KPS correlate with PROMIS Physical Function in this or previous studies?*

Unfortunately, this was a deficit in the multicenter study design and clinician-reported performance status is not available. However, we would point to prior literature showing that patient-reported performance status compares favorably to and should be used with clinician assessed performance status ^1,2^.

*•    Given that a number of other studies (reviewed in Kos et al., 2021) have demonstrated the feasibility of collecting wearable device data during routine cancer treatment and significant associations with performance status, please clarify what is the novel contribution of this manuscript to the field.*

We thank the reviewer for this reference. Kos et al indeed have an excellent review of 14 studies related to the use of fitness trackers in oncology. However, we feel this study is somewhat unique in that context:

- None of the reviewed studies were done in hematologic malignancies or include patients preparing for stem cell transplant. Those are the majority of the patients in our studies.
- Few of the referenced studies did multivariate analyses, none did the repeated measures approach. We feel these are critical components of an integrative analysis including fitness tracker data.
- Only one had tracker measured heart rate data and it wasn’t used in a model.
- None looked at the use of a tracker in the context of predicting a patient reported performance status.
- None used symptom burden in concert with a tracker in a model.
- None included concomitant exercise physiology testing.

In this context, we believe we have a novel methodological approach and a novel patient population.

In addition, we address some of the frequent shortcomings Kos et al report – namely ‘none of the studies adequately described methods used for dealing with missing physical activity data’. We put it in a heatmap to display the degree of data ‘missingness’ and then discuss how our use of summary variables and a repeated measures model mitigate this.

We would also note that 6MWT is now generated automatically by some devices (ex. the Apple iWatch), which is particularly relevant to our direct comparison of in person 6MWT to a co-occurring derived 6MWT.

*Minor Concerns
•    How did the 32 patients included in the models differ from the 12 enrolled and included in the Table 1 study cohort but who did not have sufficient data available with regard to demographics?*

We have added this information at the end of the paragraph in the results section entitled ‘Average daily activity, heart rate…’.

*•    Relatedly, any significant differences in feasibility of patient-generated data collection across the three different cohorts of patients?*

Unfortunately we do not have access to the original screening data at this level of granularity. In addition, given the small numbers of patients in some subgroups, it would likely be difficult to make broader inferences about feasibility from these data.

*•    Which result does the following statement from the Discussion refer to:  “Increased amounts of data, from both surveys and sensors, led to better predictions, as seen in our repeated measures analysis, suggesting a role for continuous, longer duration monitoring.”*

Thank you for pointing this out. We have clarified the statement to read as follows:

In addition, we found that the increased quantity of sensor and survey data provided by repeated longitudinal assessments led to better predictions, as seen in our repeated measures analysis. This suggests a role for continuous, longer duration monitoring in future studies.

*•    Why was data synchronized to the Fitbit servers only every 23 days on average?  Were patients instructed to sync the data at regular intervals?  Also the association between synchronization frequency and amount of missing data seems obvious.*

Due to the multicenter nature of the trial, most of this information is not available to us. However, we believe this may be due heterogeneity in the method of synchronization. We attempted to illustrate this in an unbiased fashion in Figure S1.

Our assumption is that some patients had the devices paired with their phones and left the app running, which likely resulted in very frequent synchronization. Others may not have had the app running continuously resulting in less frequent synchronization. Others had them paired with home computers which synchronized only when the device was physically linked. Over the duration of the study, the study team began checking verbally whether patients had synchronized their devices at their periodic calls to check in, which may have resulted in improved synchronization frequency for some.

*•    While not a stated goal of the study and based on a tiny subsample, the comparison between in-person and sensor-derived 6MWT is interesting, especially given the challenges noted in obtaining in-person 6MWT.*

We agree! And it is even more relevant now that devices like the Apple iWatch gather this automatically.

*Reviewer #2: The manuscript by Wood et al is a prospective observational feasibility study examining the use of objective data sources and structured patient generated health data tools to assess clinical performance status during routine clinical care done through the alliance. The project is in an area of high need which is to help improve objective assessments of performance status while leveraging wearables and validated surveys. The manuscript is well written and reflects on challenges encountered as well as optimum analysis of extensive data collected during the study. There are a few concerns:
A)    This is a feasibility study but the authors don’t mention any a priori parameters to indicate feasibility. They do describe low feasibility for baseline CPET and 6MWT because it was obtained only in 68% patients but don’t mention as to what the cut-off for feasibility was.*

We have now added this (see Reviewer #1, concern #1).

*B)    My other big concern with feasibility is being able to get only 44 final patients out of 54 screened patients over a course of 20 months or so from 4 large cancer centers for the study.*

We have added the a priori feasibility criteria from the study to the manuscript as noted above. The 20 month duration was likely lack of institutional prioritization / promotion of the study over others. However, this is an issue with many oncology studies not focused on an intervention. In our experience, this is solved in a multimodal fashion that is institution dependent.

*C)    More details needed about the recruitment plan- were consecutive patients approached? Were the 54 screened patients a subset of a larger group of patients who were approached but declined since their inclusion criteria seems not very restrictive? How were these 54 patients selected?*

Patients were approached at the discretion of individual providers, which likely contributed to the 20-month enrolment period. We agree that a more systematic approach would be helpful in future studies.

*D)    Not clear what is meant by ‘both cohorts’ in line 202 as in the above section, the authors mention the original cohort which was divided into 3 cohorts: solid tumor vs. heme malignancy vs. pre HCT.*

We apologize – that was a typographical error from an earlier version of the manuscript. It has been deleted.

**References**

1	Suh, S. Y., Leblanc, T. W., Shelby, R. A., Samsa, G. P. & Abernethy, A. P. Longitudinal patient-reported performance status assessment in the cancer clinic is feasible and prognostic. *J Oncol Pract* **7**, 374-381, doi:10.1200/JOP.2011.000434 (2011).

2	Popovic, G. *et al.* Patient-Reported Functional Status in Outpatients With Advanced Cancer: Correlation With Physician-Reported Scores and Survival. *J Pain Symptom Manage* **55**, 1500-1508, doi:10.1016/j.jpainsymman.2018.02.015 (2018).
