# Supplementary material for: Wearable sensor-based performance status assessment in cancer: A pilot multicenter study from the Alliance for Clinical Trials in Oncology (A19_Pilot2)
Source: PLOS Digit Health. 2023 Jan 26;2(1):e0000178. doi: 10.1371/journal.pdig.0000178 (PMC9931326; doi:10.1371/journal.pdig.0000178)
Supplement: Attachment — Submitted filename: reviewer_response plos digital health 11.20.22 upload.docx [file pdig.0000178.s012.docx]

To the Editors:

We thank the reviewers for their time and insightful comments and suggestions. Below we address the points raised by the reviewers (text indicated in blue italic font) and indicate how we have updated the manuscript.

Reviewer #1:

Consider exchanging “physical function” for “performance status” given that patient-reported physical function was the representation of performance status selected and that more traditional clinician-rated performance status measures were not assessed.

*We have included additional mentions of “physical function” in the manuscript for clarity.*

Please add a citation to support the claim that “exercise testing is the gold standard for performance status assessment.”

*We have removed the clause including the words “gold standard” as we recognize that this is a debatable claim. There are data to support the predictive and prognostic usefulness of exercise testing in a variety of cancer settings, but we did not think that the including the term “gold standard” added further value to the results we are reporting here.*

I appreciate the authors’ edits to clarify the primary feasibility aims of this paper. How was “wearing a Fitbit at least 8 hours per day” (line 157) originally operationalized? If based on something like the availability of heart rate or step data for 8 hours per day, this actually seems more stringent than “100 steps/day for two days per week” since 100 steps can be accrued in far fewer than 8 hours and two days per week seems very minimal. What is the justification for this threshold for “usable” tracker recorded step data and the decision not to focus on 8 hours per day of Fitbit wear time?

*We found it challenging to operationalize the original, though arbitrary, idea of 8 hours per day Fitbit data and opted to use as much data as possible for contribution to the models reported. We recognize that defining standards for usable tracker data will remain an evolving area for discussion in the field.*

Line 227-228 describes analytic methods for comparing baseline CPET and 6MWT and should be omitted.

*We have omitted this description.*

I appreciate the authors efforts to justify the inclusion of the sleep sensor data and self-reported sleep data but still feel this is irrelevant to the paper’s focus on performance status/physical function and should be omitted (lines 234-235 & 237-238; lines 317-322 & Supplemental Table 2).

*We would prefer to leave this material in the manuscript; we recognize that it was not part of the main focus of the study, though does provide some indirectly insightful information about physical function via impact on symptom burden and may be hypothesis-generating for others who wish to pursue this line of study further. To reflect the indirect relevance of this information, the results have been left in the supplemental data section.*

The lack of clinician-rated ECOG and KPS should be acknowledged as a limitation of this multicenter study.

*We have added an acknowledgement of this limitation to the discussion.*

Given relevance to this manuscript, the Kos et al 2021 review should be cited and included in the Introduction or Discussion.

*We have now cited and included the Kos et al 2021 review in the Discussion.*

Re: line 335, please confirm that age did not significantly differ in the included participants vs. the excluded participants.

*Age did not significantly differ in the included participants vs. the excluded participants.*

Reviewer #2: Authors have attempted to address all the comments within the constraints of the collected data. No further comments at this time

*We thank Reviewer #2 for the favorable review of this manuscript.*
